# Random and aligned electrospun PLGA nanofibers embedded in microfluidic chips for cancer cell isolation and integration with air foam technology for cell release

**DOI:** 10.1186/s12951-019-0466-2

**Published:** 2019-02-19

**Authors:** Chia-Cheng Yu, Yi-Wen Chen, Po-Ying Yeh, Yu-Sheng Hsiao, Wei-Ting Lin, Chiung-Wen Kuo, Di-Yen Chueh, Yun-Wen You, Jing-Jong Shyue, Ying-Chih Chang, Peilin Chen

**Affiliations:** 1 Department of Materials Engineering, Ming Chi University of Technology, Taishan, New Taipei City, 24301 Taiwan; 2 Research Center for Applied Sciences, Academia Sinica, Taipei, 11529 Taiwan; 3 Genomics Research Center, Academia Sinica, Taipei, 11529 Taiwan

**Keywords:** Circulating tumor cells, Poly(lactic-*co*-glycolic acid) (PLGA), Nanofiber arrays, Air foam

## Abstract

**Background:**

Circulating tumor cells (CTCs) comprise the high metastatic potential population of cancer cells in the blood circulation of humans; they have become the established biomarkers for cancer diagnosis, individualized cancer therapy, and cancer development. Technologies for the isolation and recovery of CTCs can be powerful cancer diagnostic tools for liquid biopsies, allowing the identification of malignancies and guiding cancer treatments for precision medicine.

**Methods:**

We have used an electrospinning process to prepare poly(lactic-*co*-glycolic acid) (PLGA) nanofibrous arrays in random or aligned orientations on glass slips. We then fabricated poly(methyl methacrylate) (PMMA)-based microfluidic chips embedding the PLGA nanofiber arrays and modified their surfaces through sequential coating with using biotin–(PEG)_7_–amine through EDC/NHS activation, streptavidin (SA), and biotinylated epithelial-cell adhesion-molecule antibody (biotin-anti-EpCAM) to achieve highly efficient CTC capture. When combined with an air foam technology that induced a high shear stress and, thereby, nondestructive release of the captured cells from the PLGA surfaces, the proposed device system operated with a high cell recovery rate.

**Results:**

The morphologies and average diameters of the electrospun PLGA nanofibers were characterized using scanning electron microscopy (SEM) and confocal Raman imaging. The surface chemistry of the PLGA nanofibers conjugated with the biotin–(PEG)_7_–amine was confirmed through time-of-flight secondary ion mass spectrometry (ToF–SIMS) imaging. The chip system was studied for the effects of the surface modification density of biotin–(PEG)_7_–amine, the flow rates, and the diameters of the PLGA nanofibers on the capture efficiency of EpCAM-positive HCT116 cells from the spiked liquid samples. To assess their CTC capture efficiencies in whole blood samples, the aligned and random PLGA nanofiber arrays were tested for their abilities to capture HCT116 cells, providing cancer cell capture efficiencies of 66 and 80%, respectively. With the continuous injection of air foam into the microfluidic devices, the cell release efficiency on the aligned PLGA fibers was 74% (recovery rate: 49%), while it was 90% (recovery rate: 73%) on the random PLGA fibers, from tests of 200 spiked cells in 2 mL of whole blood from healthy individuals. Our study suggests that integrated PMMA microfluidic chips embedding random PLGA nanofiber arrays may be suitable devices for the efficient capture and recovery of CTCs from whole blood samples.

**Electronic supplementary material:**

The online version of this article (10.1186/s12951-019-0466-2) contains supplementary material, which is available to authorized users.

## Background

Prior to its diagnosis, cancer malignancy begins with the invasion of motile cancer cells into the circulatory system [1]. In recent years, many researchers have been searching for ways to identify these rare cells in the circulatory systems through regular liquid biopsies that include the collection of small amounts of specific cancer cells from blood vessel systems [2]. To date, however, only the CellSearch System—using microbeads magnetically labeled with epithelial cell adhesion molecule antibodies (anti-EpCAM)—has been approved by the US Food and Drug Administration (FDA) for the enrichment of circulating tumor cells (CTCs) during liquid biopsies [3]. Because the structural heterogeneity of CTCs makes it difficult to capture them during their circulation in the blood, the ability to do so would presumably be of major interest in the fields of cancer biology and biomedical engineering.

Integrating bio-responsive materials with polymers through surface modification, top-down structural design, and molecularly based cascade structures should lead to many new applications in the upcoming years [4]. In the field of polymer processing and manufacturing, the capture of CTCs became possible after the discovery that skeletal structures having very narrow dimensions (e.g., nanofibers) could be integrated with the extracellular matrices of cells [4, 5]. Such nanoscale fibers can be spun using high voltage saturation, in a process known as electrospinning, which has been gaining increasing attention in applied medical and biomedical engineering [6]. Selecting the compounds most suitable for electrospinning remains a challenge; at present, raw polymers are employed predominantly, but the use of surface-modified polymers is growing. The most important polymers [7–13] include polyurethane, polybenzimidazole, polycarbonate, polyacrylonitrile, poly(vinyl alcohol), poly(lactic acid), poly(ethylene-*co*-vinyl acetate), poly(ethylene oxide), collagen, polyaniline, and poly(ethylene glycol); among them, silk, chitosan, poly(ethylene glycol) and collagen, as well as poly(lactic-*co*-glycolic acid) (PLGA) [14–18], have been attracting growing interest as biocompatible polymers for the preparation of nanofibers for the capture of rare cancer cells [19–22].

PLGA is one of the most attractive biocompatible polymers; it has been approved by the FDA and is used generally in the fabrication of nanofibers [23]. Recent progress in the fabrication of PLGA nanofiber arrays has led to many biomedical applications, including guided tissue regeneration [7, 8, 17], enhanced human endometrial-derived stromal cell (hEnSC) proliferation in cellular therapy [24], potential local chemotherapy for breast tumor formation [25], and the isolation of CTCs through the NanoVelcro cell‐affinity assay [26–29].

Because PLGA materials feature terminal carboxyl groups, they can undergo surface modification with amine-based compounds [through *N*-ethyl-*N*′-(3-(dimethylamino)propyl)carbodiimide/*N*-hydroxysuccinimide (EDC/NHS) coupling] and can be conjugated with streptavidin (SA) for biotin-based affinity experiments. Improving the surface chemistry of PLGA nanofiber structures can help in the isolation of CTCs from whole blood samples [30]. In this study, we prepared PLGA nanofibers with random and aligned morphologies on glass cover slips. After incorporating an anti-EpCAM antibody coating on these nanofibers, we assembled them into poly(methyl methacrylate) (PMMA)-based microfluidic devices and compared the ability of the random and aligned electrospun PLGA nanofibers to capture EpCAM-positive HCT116 cancer cells from whole blood samples. We also evaluated the integration of this system with an air foam technology for release of the captured cells.

## Experimental section

### PLGA nanofibrous arrays

**Fig. 1:**
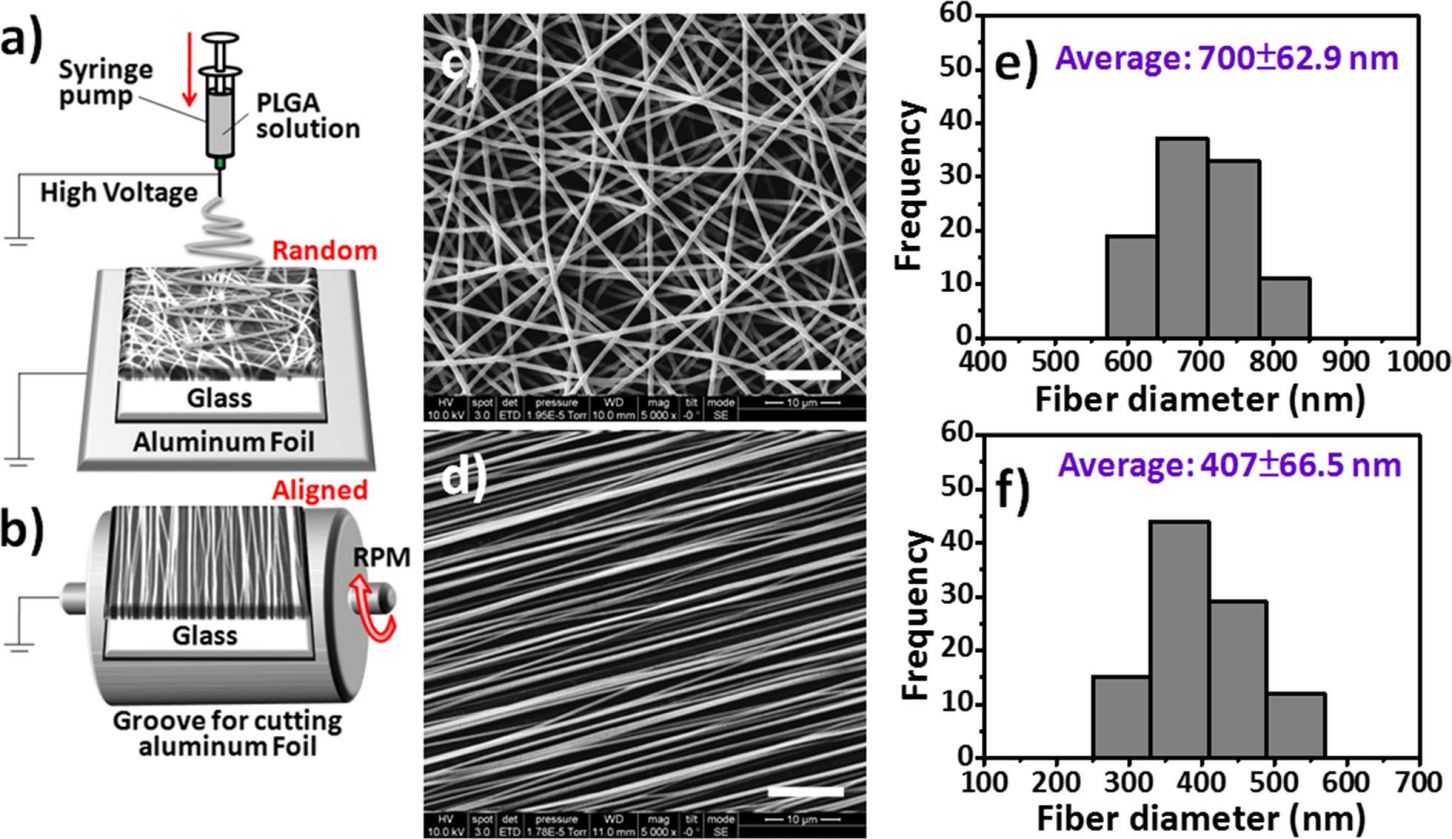
**a**, **b** Schematic representation of the setups for electrospinning **a** random PLGA nanofiber arrays on a stationary glass cover slip and **b** aligned PLGA nanofiber arrays collected on a glass cover slip when using a spinning drum. **c**, **d** SEM images and **e**, **f** diameter distribution histograms of the **c**, **e** random and **d**, **f** aligned PLGA nanofiber arrays

The 50/50 PLGA with carboxyl end groups was purchased from LACTEL Absorbable Polymers (Cupertino, CA, USA; lactide/glycolide, 50/50; inherent viscosity range, 0.55–0.75 dL g^−1^). 1,1,1,3,3,3-Hexafluoro-2-propanol (TFIP, 99%) was purchased from Sigma-Aldrich and used without purification. PLGA (1.0 g) was dissolved in HFIP (9 mL) through stirring at room temperature on a magnetic stirrer for 6 h, at which point the solution became homogeneous and transparent. This PLGA solution was loaded in a 10-mL syringe and capped with a fine 27-gauge needle. Electrospinning is performed in an ambient environment at a relative humidity of less than 40%. To optimize the PLGA nanofibrous arrays with suitable size distributions, thicknesses, and surface morphologies for CTC capture and isolation, the following fabrication parameters were tested: the concentration of the PLGA solution (5–20 wt%), the electrical potential (10–20 kV), the distance between the injection needle and ground collector (15–20 cm), and the feeding rate (0.1–0.5 mL h^−1^). The random nanofiber arrays were collected on a flat collector plate wrapped with aluminum foil, as illustrated in Fig. 1a. The aligned nanofibers were formed using a rotating drum setup operated at 3000 rpm, as illustrated in Fig. 1b. Both the random and aligned nanofibers were collected on 24 mm × 50 mm glass cover slips. The resulting PLGA nanofibrous array–coated cover slips were vacuum-dried for 24 h to eliminate any residual organic solvent.

### Characterization of PLGA nanofibrous arrays

The diameters, distributions, and orientations of PLGA nanofibers were characterized through scanning electron microscopy (SEM), using an FEI Nova NanoSEM 200 instrument operated at an accelerating voltage of 10 keV. The PLGA samples for SEM imaging were sputter-coated with gold (< 3 nm). The morphological and chemical structures were characterized using micro-Raman spectrometry (Alpha 300, WITec Instruments, Germany; resolution: 1 cm^−1^; laser excitation: 514.5 nm), with observation of the Raman shifts in the range from 500 to 2000 cm^−1^ [31, 32]. Two-dimensional (2D) Raman mapping images of the random/aligned PLGA nanofiber arrays were recorded according to the intensity of the noticeable band at 1760 cm^−1^, which represented the ester linkages of PLGA. The surface modification of the PLGA nanofibers with biotin–(PEG)_7_–amine (MW = 550; Laysan Bio, Arab, Alabama), mediated through EDC/NHS chemistry, was analyzed using advanced time-of-flight secondary ion mass spectrometry (ToF–SIMS) characterization and imaging. ToF–SIMS experiments were performed using a PHI TRIFT V nanoTOF (Chigasaki, Japan) ToF–SIMS system. The reticulated PLGA nanofiber arrays were electrospun onto Al foil prior to their transfer into the system for analysis [33]. The details regarding the ToF–SIMS characterization are described in Additional file 1. The topographies of the PLGA nanofibers were analyzed using atomic force microscopy (AFM); the Bruker Dimension Edge microscope was operated in tapping mode at ambient temperature.

### Device assembly, surface engineering, and cell capture and release tests

The PLGA nanofiber array-coated glass cover slip (bottom) was bonded with a PMMA top plate (area: 25.4 mm × 76 mm; thickness: 1.5 mm) through sandwiching of laser-engraved double-side acrylic adhesive tape (8018PT; 3M, Maplewood, MN) to form microfluidic channels with appropriate sealing. The dimensions of the finished device were equal to those of a standard microscope slide, allowing ease of direct observation under an inverted microscope system. To fabricate the PMMA top channel plate, a commercial CO_2_ laser engraving system (Universal VLS2.30, Universal laser System, AZ, USA) with high-power density focusing optics (HPDFO) was used to drill two holes (as the inlet and outlet of the microfluidic chip) and engrave the microstructures on the PMMA plate (the dimensional design of the chaotic mixing module of the chip has been described previously [34]). The device had pocket microstructures that were 250 µm wide and 50 µm deep, with a channel height of 60 µm (Fig. 5a, b). Two PMMA connectors, bonded to the PMMA top channel plate by applying chloroform to the interface, were used to join the outer tubes into the inner micro-channels. For surface engineering of the PLGA nanofibrous arrays, a three-step coating sequence was employed to link biotin–(PEG)_7_–amine, SA, and biotinylated anti-EpCAM to the carboxylic acid-terminated PLGA nanofiber surfaces. In this manner, the surface chemistry of the PLGA materials was made suitable for use in the isolation of CTCs [20, 35]. In brief, the carboxylate end groups of the PLGA nanofibers were subjected to EDS/NHS coupling chemistry [36, 37] to form peptide bonds with biotin–(PEG)_7_–amine, thereby providing binding sites for the sequential coating of SA [10 μg mL^−1^ in 1× phosphate-buffered saline (PBS) for 1 h] and the biotinylated anti-human EpCAM/TROP1 antibody [10 μg mL^−1^ in 1× PBS with 0.1% bovine serum albumin (BSA) for 1 h] through biotin–SA conjugation [38] for the capture of CTCs through a controlled immunoaffinity procedure [39]. EDC was obtained from TCI (Tokyo, Japan); NHS was obtained from Sigma (St. Louis, MO, USA); SA and biotinylated anti-human EpCAM/TROP1 antibody (Goat IgG) were obtained from R&D Systems (Minneapolis, MN, USA). In the cell capture/release tests, the injection flow rates in the microfluidic channels were controlled from 1 to 10 mL h^−1^. The captured cells were carefully recovered through the continuous injection of air foam into the microfluidic devices, using a cell-release method described previously [40].

### Cell culture

Various cells were used to check the performance of the microfluidic devices and optimize their capture and release of CTCs: human colon cancer cells (HCT116), breast cancer cells (MCF7), cervical cancer cells (HeLa), and monocytic cells (THP1), all purchased from Bioresource Collection and Research Center (BCRC, Taiwan). The lung adenocarcinoma cancer cell line (PC9) was kindly gifted by Prof. Sung-Liang Yu (Department of Clinical Laboratory Sciences and Medical Biotechnology, College of Medicine, National Taiwan University, Taiwan). The human hepatoma cancer cell lines (HepG2 and Huh7) were kindly gifted by Prof. Kin Fong Lei (Graduate Institute of Biomedical Engineering, Chang Gung University, Taiwan). The HCT116, MCF7, PC9, HepG2, and Huh7 cells were used as EpCAM-positive cell lines; the HeLa and THP1 cells were used as EpCAM-negative cell lines. These cells were reviewed and used in the present study through growth in Dulbecco’s modified Eagle medium (DMEM; Gibco, Invitrogen, Carlsbad, CA) containing 10% fetal bovine serum (FBS, HyClone, Australia) and 1% antibiotic–antimycotic (Invitrogen Life Technologies, Carlsbad, CA, USA) under an environment of 5% CO_2_ at 37 °C. Prior to application of the cells, 0.25 M trypsin was applied for trypsinization of the cells; the resulting cells were re-suspended in the fresh media at a desired concentration. The HCT116 cells were pre-stained with CellTracker Green CMFDA dye (Invitrogen Life Technologies, Carlsbad, CA, USA) prior to performing spiking experiments.

### Cell staining and identification

The cell viabilities and cell counts for dilution or cell spiking were assessed conventionally using a Luna cell counter (Logos Biosystems, South Korea). For artificial blood tests, the captured cells were treated through immunofluorescence staining to identify and enumerate the cell types on the PLGA nanofibers. 4′,6-diamidino-2-phenylindole (DAPI) (Invitrogen Life Technologies, Carlsbad, CA, USA) was used to stain the nuclei of all cells; rabbit antihuman cytokeratin 20 (CK20; Abcam, Cambridge, UK) was employed to stain the HCT116 cells; rat anti-human CD45 (Abcam, Cambridge, UK) was employed to stain the white blood cells (WBCs) overnight at 4 °C. After 1× PBS washing, the cells were incubated with FITC-conjugated goat anti-rat IgG antibodies (Abcam, Cambridge, UK) and Alexa Fluor1 568 anti-rabbit IgG antibodies (Invitrogen Life Technologies, Carlsbad, CA, USA) for 1 h at room temperature, followed by washing with 1× PBS. The cells were then imaged immediately using a fluorescence confocal microscope (Olympus FV 10i, Japan).

### Statistical analysis

All experiments were performed at least three times for each sample; mean values (E standard deviation) are reported.

## Results and discussion

### Electrospinning of PLGA nanofibrous arrays

Fiber-based scaffolds can be generated from many natural or synthetic polymers; they structurally mimic the environment in the extracellular matrix (ECM) and, thereby, ensure more efficient cell–substrate interactions than those provided by planar structures [30, 41]. When combined with suitable cell capture agents [e.g., epithelial cell adhesion molecule antibodies (anti-EpCAM)], the nanostructured surfaces can provide a synergistic effect for enhanced CTC isolation [42–46]. In this context, electrospun nanofibrous scaffolds not only mimic the nano-sized dimensions of the natural ECM with spatial organization on the mesoscopic scale (control over fiber orientation, packing density, and spatial placement) but also provide a simple approach toward engineering nanostructured surfaces to present various bioaffinity agents. Therefore, in this study, we fabricated and characterized both random and aligned electrospun nanofibrous arrays of PLGA, a synthetic biodegradable polymer, and evaluated their CTC capture efficiencies; we also investigated the cell-release performance through the continuous injection of air foam into the PMMA microfluidic devices. We chose PLGA for this study because it is readily formed into desired shapes with good mechanical strength, has a suitable degradation time scale, and has outstanding biocompatibility. The electrospinning of the random and aligned PLGA nanofibers arrays is illustrated in Fig. 1a, b, respectively. The resultant nanofibrous arrays (solution concentration, 10 wt%; electrical potential, 15 kV; collection distance, 15 cm; feeding rate, 0.1–0.5 mL h^−1^) had a random morphology (Fig. 1c, e), as observed using FE-SEM with a fiber diameter distribution of 700 ± 63 nm, or an aligned morphology with a remarkably different diameter distribution of 407 ± 67 nm (Fig. 1d, f), when formed on a drum rotating at a constant rate of 3000 rpm. When the speed of the rotating mandrel was varied from 1000 to 3000 rpm, most of the resulting nanofibers exhibited a longitudinal orientation, due to the match between the speed of the rotating mandrel and the rate at which the fibers were deposited on the collector. Accordingly, the diameter distributions of the fibers produced under the various rotation rates was centered approximately between 300 and 500 nm (Fig. 1f). Since Yu et al. [47] found that a high speed rotating drum as a collector would result in a drafting force for stretching nanofibers, thereby decreasing the diameter and alignment degree of electrospun nanofibers. Therefore, it is reasonable to infer that the decreasing diameter of PLGA nanofibers with increase of the drum rotation speed might be due to the drafting force of the rotating drum in our experiments. Among the investigated fabrication parameters, the concentration of the PLGA solution has the greatest effect on the fiber diameter, followed by the feed rate; the electric field strength was the least influential factor. Therefore, using these findings, we could readily tailor the surface modification of the PLGA nanofiber arrays to optimize the cell-capture efficiency in our subsequent experiments.

### Surface characterization

**Fig. 2:**
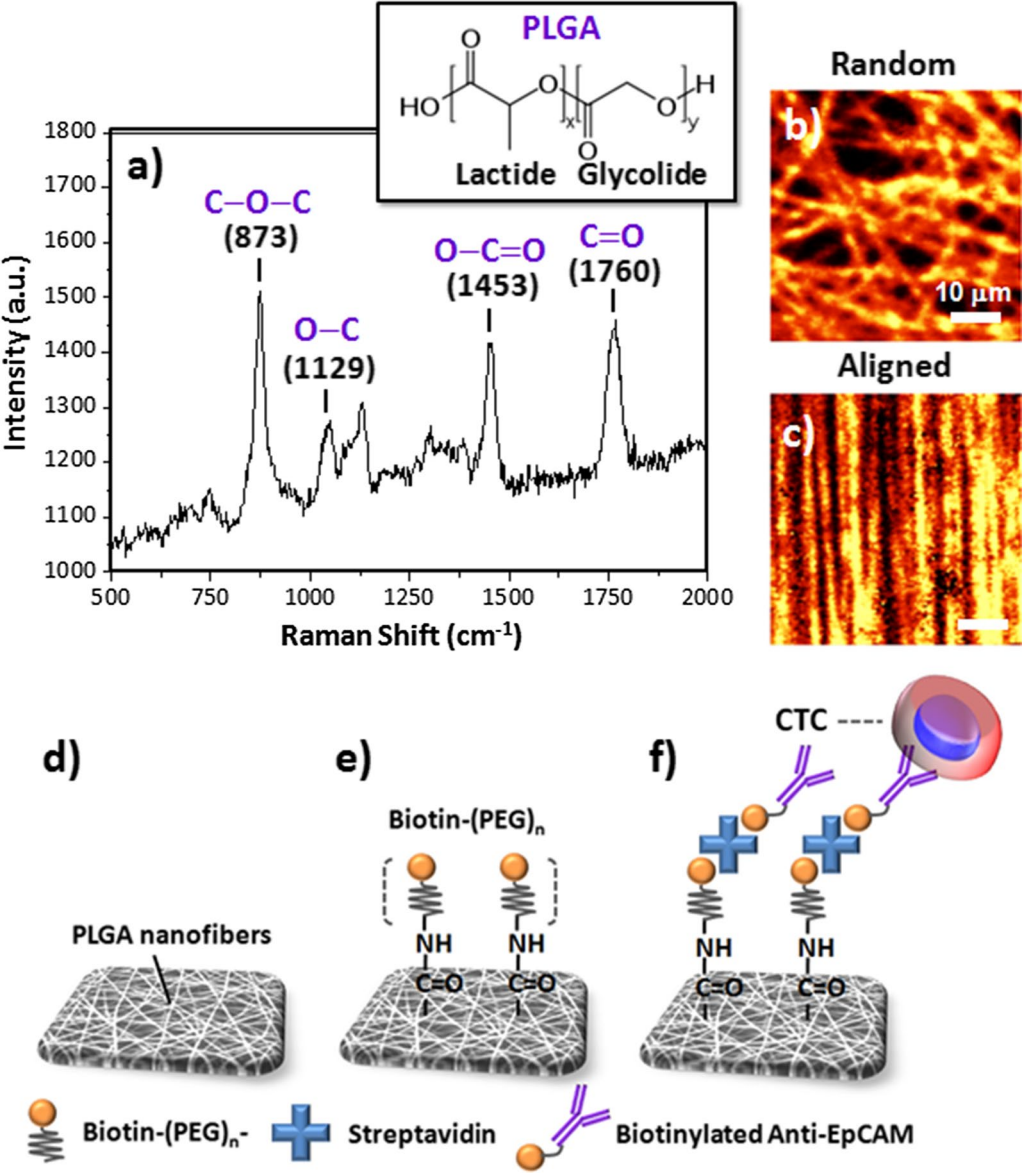
**a** Compositional analysis of the PLGA nanofibers through Raman spectroscopy, with bond stretching of C–O–C units at 873 cm^−1^, O–C at 1129 cm^−1^, O–C=O at 1453 cm^−1^, and C=O at 1760 cm^−1^. **b**, **c** Confocal Raman images of the random and aligned PLGA nanofiber arrays, according to the intensities of their signals at 1760 cm^−1^. **d**–**f** Schematic representations of the bioconjugation of biotinylated anti-EpCAM to PLGA nanofiber arrays for CTC isolation: **d** morphology of the nanofibers from SEM imaging; **e** EDC/NHS-mediated bonding of the biotin–(PEG)_*n*_ to the surface of the PLGA nanofibers; **f** SA bonding to form biotinylated anti-EpCAM antibodies for capturing CTCs

To examine their compositions and morphologies, we recorded the Raman spectra of the random and aligned PLGA nanofibrous arrays after excitation at 514.5 nm (Fig. 2a–c) [31, 32]. We attribute the strong characteristic bands near 1760 cm^−1^ to the stretching vibrations of the ester C=O bonds, the bands at 1180 cm^−1^ to stretching of the C–O–C ether groups, the bands at 873 cm^−1^ to the O–C=O stretching modes of the lactic acid units, and the bands at 1129 and 1453 cm^−1^ to the C–O and methyl C–H bonds of PLGA, respectively. These signals are consistent with those reported previously [16, 18]. After mapping the 2D Raman images of the PLGA nanofibers having the randomly and aligned orientations, according to the intensities of their signals at 1760 cm^−1^, the observed PLGA nanofibers arrays (Fig. 2b, c, respectively) featured the same structures as those observed in the SEM images. To conjugate the PLGA nanofibers with a specific anti-EpCAM for the CTC isolation, we conducted a three-step coating sequence involving the binding of biotin–(PEG)_7_–amine, SA, and biotinylated anti-EpCAM antibodies to the carboxylic acid-terminated PLGA nanofiber surfaces (Fig. 2d–f). The covalent bonding of biotin–(PEG)_7_–amine to the surface of the PLGA nanofibers occurred through EDC/NHS coupling between the carboxylic acid and amino groups; the subsequent binding of SA and the biotinylated anti-EpCAM antibodies both occurred through noncovalent biotin–SA interactions. We suspected that the final immunoaffinity-modified PLGA nanofibers would provide a synergistic capture strategy for enhancing CTC isolation, relative to those of the antibodies without the nanostructures and the nanostructures without the antibodies.

**Fig. 3:**
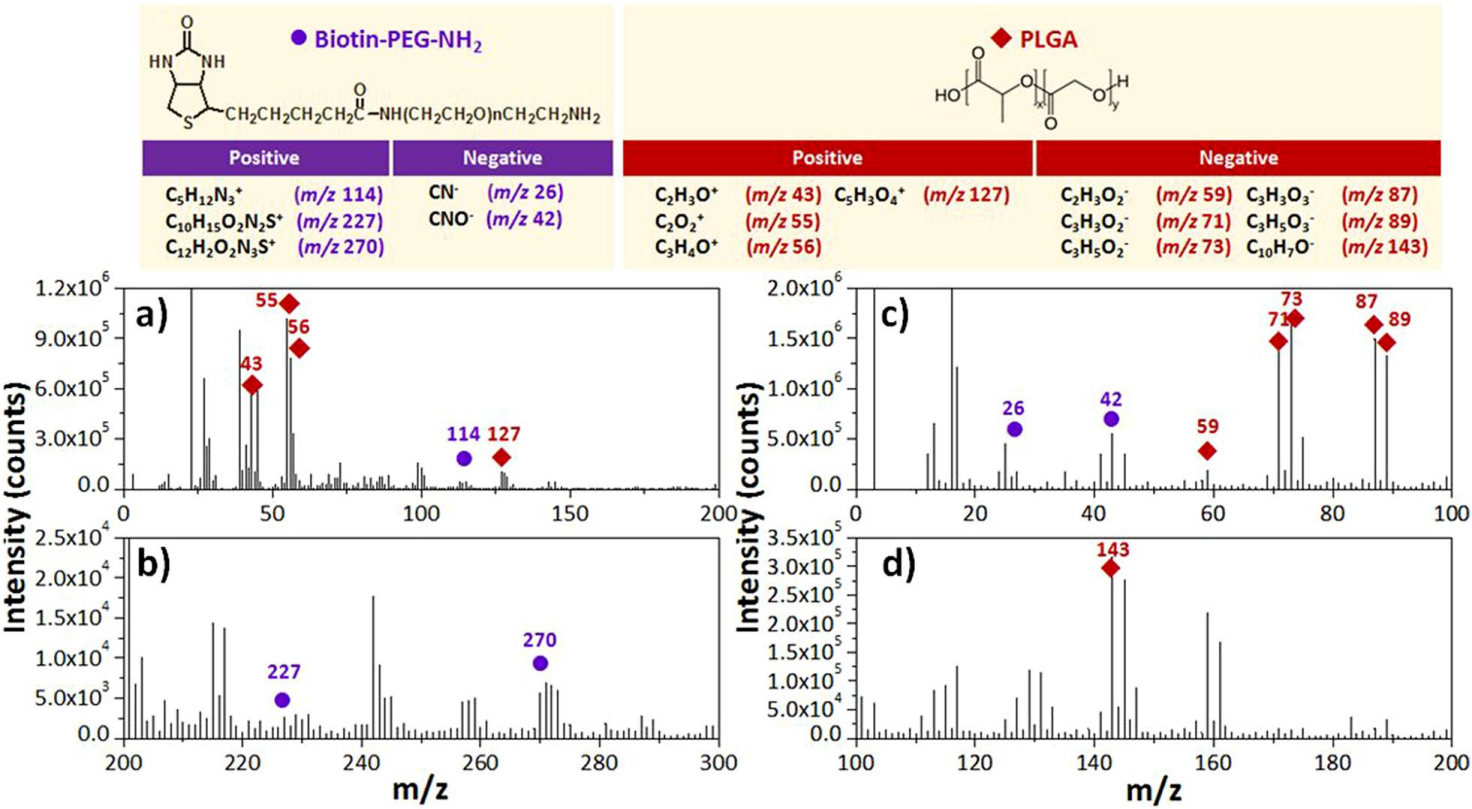
**a**, **b** Positive and **c**, **d** negative ToF–SIMS spectra of PEGylated biotin-conjugated PLGA nanofibers

**Fig. 4:**
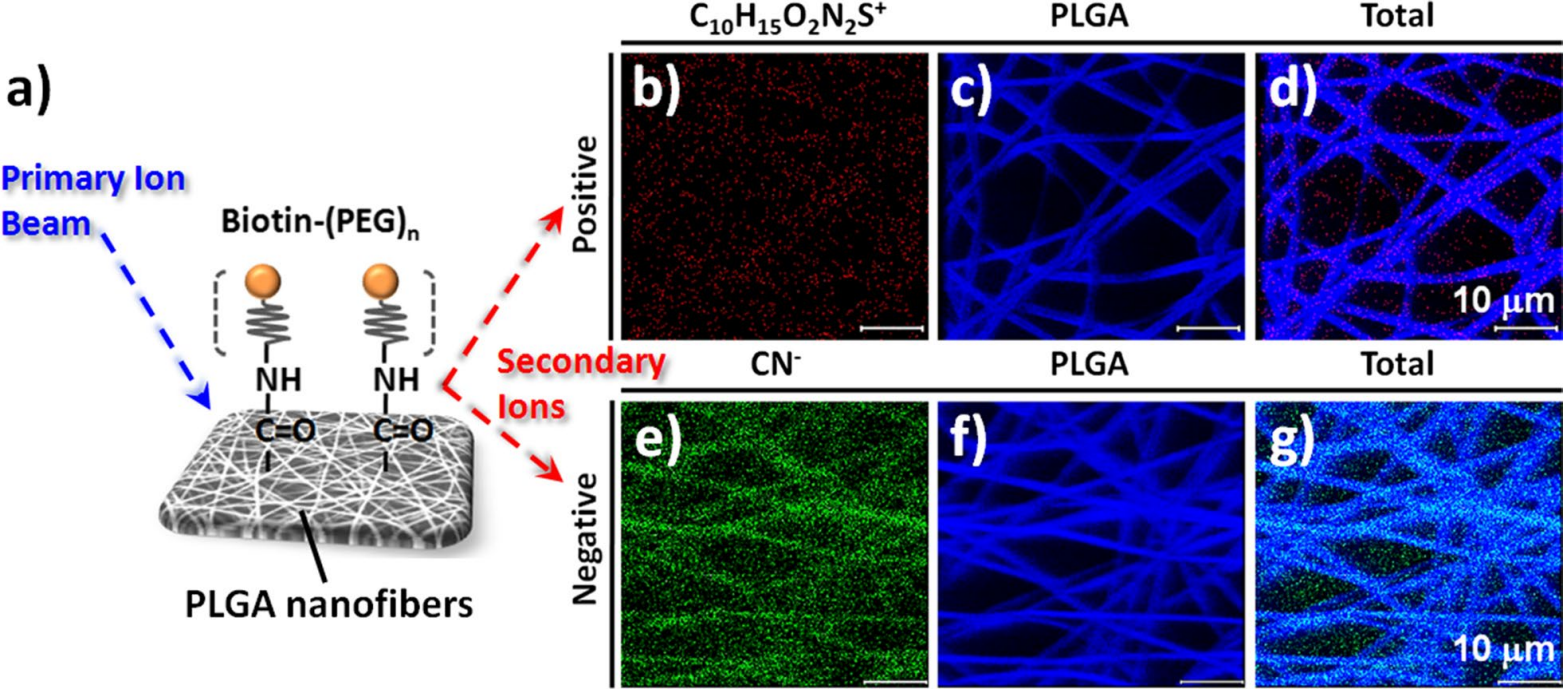
**a** Schematic representation of the conjugation of PEGylated biotin on the surface of the PLGA nanofiber arrays for ToF–SIMS chemical imaging. **b**–**g** ToF–SIMS chemical images of PEGylated biotin-conjugated PLGA nanofibers in **b**–**d** positive ion mode for **b** C_10_H_15_O_2_N_2_S^+^, **c** PLGA, and **d** total ions and **e**–**g** negative ion mode for **e** CN^−^, **f** PLGA, and **g** total ions

To confirm the conjugation and spatial distribution of biotin–(PEG)_7_–amine on the PLGA nanofibers through EDC/NHS coupling, we conducted a ToF–SIMS surface analysis. This method of mass analysis has a low detection limit and high spatial resolution, thereby allowing identification of the compositions of material surfaces [48]. Figures 3 and 4 present the spatial and surface distributions of biotin–(PEG)_7_–amine on the PLGA nanofibers, as explored through ToF–SIMS spectroscopy in positive and negative ion modes. Based on the intensity counts, the ToF–SIMS spectra confirmed the conjugation of biotin–(PEG)_7_–amine to the surface of the PLGA nanofibers arrays. We assigned the characteristic signals from the secondary ions of biotin–(PEG)_7_–amine, with values of *m*/*z* of 26, 42, 114, 227, and 270, to the ions CN^−^, CNO^−^, C_5_H_12_N_3_^+^, C_10_H_15_O_2_N_2_S^+^, and C_12_H_2_O_2_N_3_S^+^, respectively (Fig. 3). In contrast, the major signals of PLGA appeared at *m*/*z* 43 (C_2_H_3_O^+^), 55 (C_2_O_2_^+^), 56 (C_3_H_4_O^+^), 59 (C_2_H_3_O_2_^−^), 71 (C_3_H_3_O_2_^−^), 73 (C_3_H_5_O_2_^−^), 87 (C_3_H_3_O_3_^−^), 89 (C_3_H_5_O_3_^−^), 127 (C_5_H_3_O_4_^+^), and 143 (C_10_H_7_O^−^) (Fig. 3). Figure 4a also illustrates the binding structures of biotin–(PEG)_*n*_ on the PLGA nanofiber surfaces. The data in Fig. 4b–g confirmed that the PLGA nanofibers provided signals for the positive and negative ions of C_10_H_15_O_2_N_2_S^+^ and CN^−^ from biotin–(PEG)_7_–amine, respectively; they were generally present in the mapping as the characteristic signals in the ToF–SIMS images. Based on the optimized conditions for conjugating biotin–(PEG)_7_–amine to PLGA nanofibers, we expected the capture of specific CTCs would be facilitated when using biotinylated antibody-modified [e.g., anti-epithelial cell adhesion molecule (anti-EpCAM)] surfaces, which are well-established immunomarkers for CTC isolation (Fig. 2f) [20, 35].

**Fig. 5:**
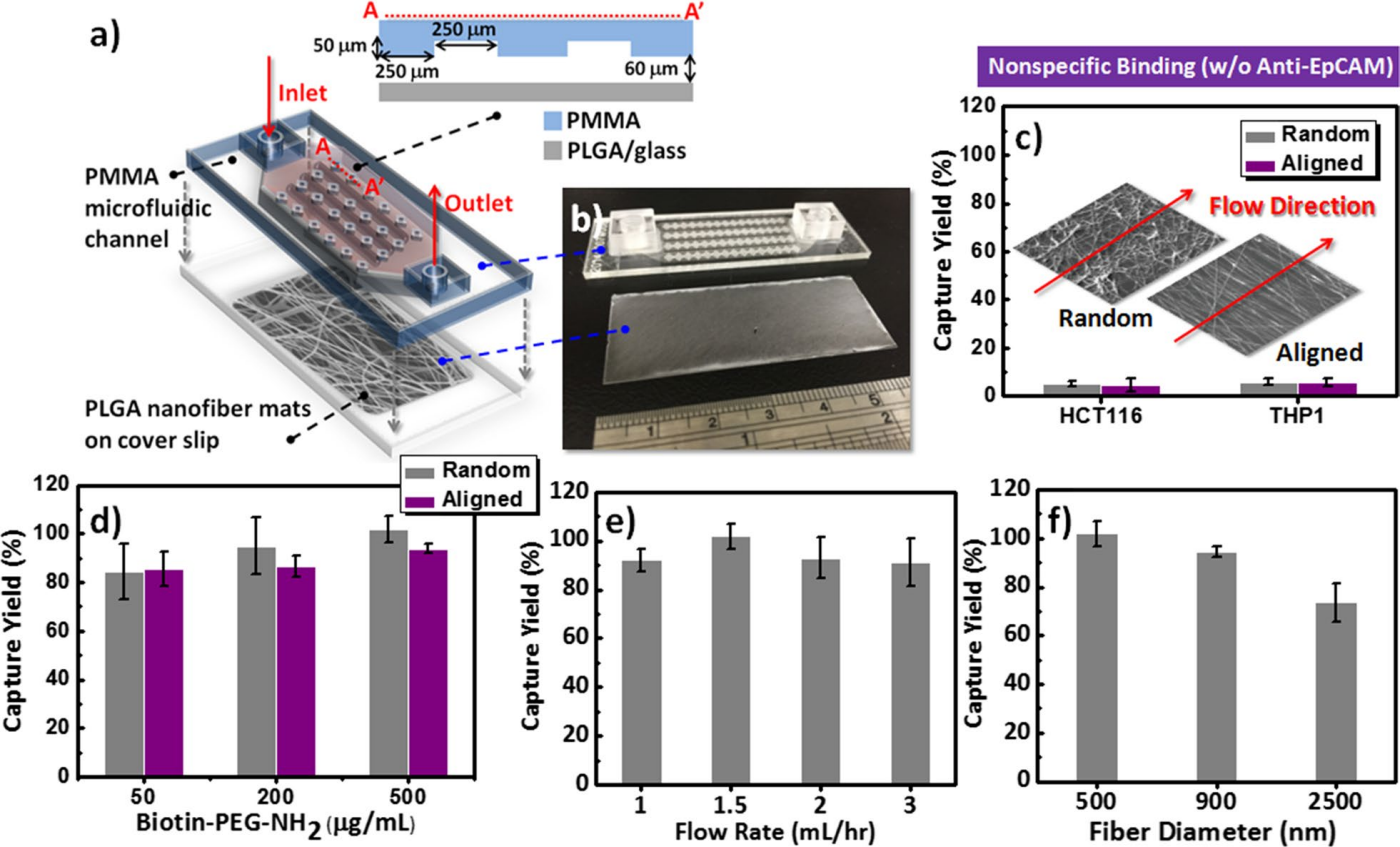
**a** Schematic representation and **b** photograph of the microfluidic device for capturing and releasing CTCs, consisting of a PLGA nanofiber array–coated glass cover slip (as the NanoVelcro bottom substrate) and a PMMA top plate (as the chaotic mixing module). **c** Performance observed for the prevention of nonspecific cell adhesion (EpCAM-positive HCT116 and EpCAM-negative THP1) on random and aligned PLGA nanofiber arrays (without anti-EpCAM coating). **d**–**f** Optimization of operational parameters for purifying CTCs: Cell-capture performance **d** when using various concentrations of biotin–(PEG)_7_–amine (50, 200, and 500 µg mL^−1^) on random and aligned PLGA nanofiber arrays (with anti-EpCAM coating) under a flow rate of 1.5 mL h^−1^; **e** at various flow rates (1, 1.5, 2, and 3 mL h^−1^); and **f** at various diameter distribution of PLGA fiber arrays (500, 900, and 2500 nm)

During recent years, many efforts have been devoted to the development of technologies for the capture and identification of rare cells, including CTCs, and fetal nucleated red blood cells [49–51]. Apart from the development of standard requirements for high capture efficiency, a challenge for these promising platforms is the release and/or recovery of the captured target cells with biological activity and, thereby, their use in downstream molecular characterization or cultivation. In previous studies, we determined that the geometry and patterned design of a PMMA microfluidic device featuring four parallel channels was suitable for maximizing the cell capture efficiency; further integration with the injection of a gentle sweep of hydrophobic air foam was sufficient to optimize the cell recovery from chips coated with an antibody-conjugated supported lipid bilayer [40]. To explore the possibility of using PLGA nanofibrous arrays for CTC capture and recovery on-chip, we applied our previous PMMA microfluidic device configuration to our present PLGA nanofiber arrays-coated system (Fig. 5a, b). We optimized the cell-capture efficiency of the devices by using the red fluorescence protein (RFP) ectopically expressed colorectal cancer cell line HCT116; this approach allowed us to demonstrate the advantages of our PLGA nanofiber-based devices in CTC liquid biopsies for personalized cancer diagnostics, with cell mixture suspensions in whole blood samples passing through the devices and monitored based on the number of spiked cells captured. The cancer cell capture yield is defined herein as the ratio of the number of HCT116 cells bound on the chip to the number of cells injected into the chip. As displayed in Fig. 5c, we initially used the EpCAM-positive HCT116 cells and EpCAM-negative THP1 leukemia cell suspensions (10^5^ cells mL^−1^ in cell culture medium) for dynamic cell-capture studies using the device systems featuring the random and aligned PLGA nanofiber arrays. Our cell-capture results were consistent with previous reports, but with extremely low nonspecific backgrounds of the EpCAM-positive or EpCAM-negative cells [30], presumably because the carboxylic acid termini of the PLGA materials resisted cell adhesion once treated with pH-8.4 phosphate-buffered saline (PBS).

As presented in Fig. 2d–f, our present device configuration involved a three-step coating sequence—biotin–(PEG)_7_–amine, SA, and biotinylated anti-EpCAM antibodies—on the carboxylic acid-terminated PLGA nanofiber surfaces, providing a means for specific binding of CTCs. Thus, we explored the effects of the surface modification conditions of the PLGA nanofiber arrays (random and aligned), namely the concentration of biotin–(PEG)_7_–amine, the flow rate, and the fiber diameters, to optimize the cell-capture efficiency. Figure 5d presents the capture yields of the HCT116 cell suspension (10^5^ cells mL^−1^ in cell culture medium) when passed through the microfluidic channels at a flow rate of 1.5 mL h^−1^. The capture yield was maximized on the random fibers at a concentration of biotin–(PEG)_7_–amine (ca. 500 µg mL^−1^) higher than that of the aligned nanofibers. In previous studies, we found that the flow rate must generally be maintained at less than 4 mL h^−1^ to increase the binding efficiency of the cells and avoid any potential loss of captured CTCs. Figure 5e displays the cell-capture performance of the microfluidic device operated at flow rates of 1, 1.5, 2, and 3 mL h^−1^; the data suggest that the optimized capture yield occurred on the random nanofibers when the flow rate was 1.5 mL h^−1^. Figure 5f displays the capture sensitivity based on the diameter of the PLGA fibers; the results suggest that smaller fiber diameter distributions resulted in higher cell-capture yields when using this device platform. Static cell-capture results confirmed that the random morphology of our PLGA nanofiber arrays was optimal: it displayed a positive response toward the capture of EpCAM-positive cancer cells (e.g., HCT116, MCF7, PC9, HepG2, and Huh7 cell lines), while resisting the adhesion of EpCAM-negative cells (e.g., HeLa and THP1 cell lines) (Additional file 1: Figure S1). However, the corresponding lower cell-capture efficiencies of HepG2 cell line than other EpCAM-positive cancer cells may be attributed to the lower expression of EpCAM protein level in HepG2 cells [52].

**Fig. 6:**
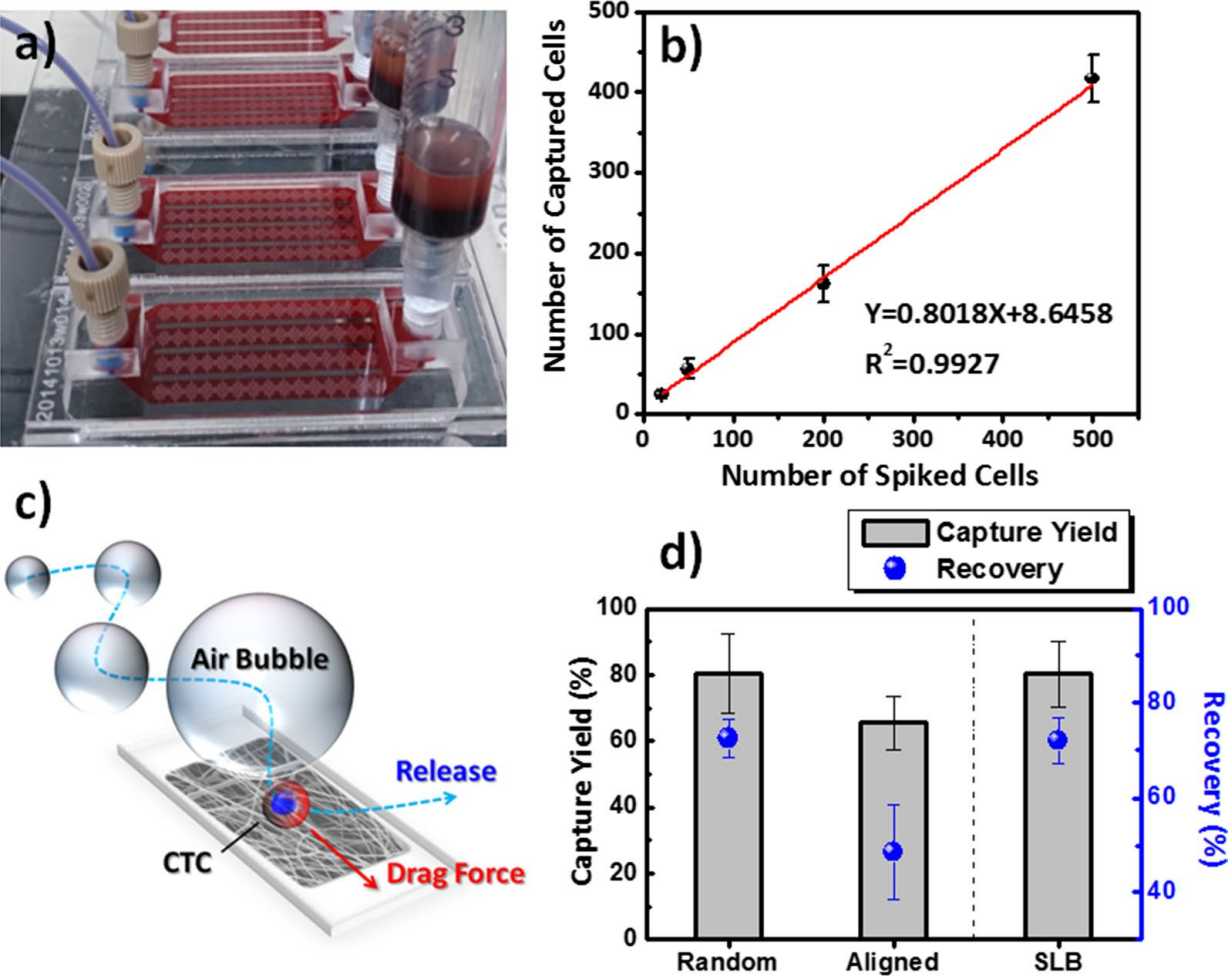
**a** Photograph of microfluidic device featuring PLGA nanofiber arrays for on-chip rare cancer cell isolation and collection from whole blood samples. **b** Cell-capture performance measured at various contents of HCT116 cells in whole blood. **c** Schematic representation of the microfluidic device system with integration of air foam technology for cell release. **d** Capture and recovery yields of the microfluidic device with antibody-conjugated PLGA nanofiber arrays or a supported lipid bilayer (SLB) for EpCAM-positive HCT116 cells

Several techniques are available for the recovery of cells intact from microfluidic devices [43–45, 49, 50]. Here, we employed an air foam approach—using a mixture of air and cell culture medium containing bovine serum albumin (BSA)—for the efficient release of the captured cancer cells from our microfluidic devices (Fig. 6). We produced the air foam solution from a mixture of air and 5% BSA that was gently vortexed for at least 1 min to create a stable foam. Although we have published this air foam technology previously, as a means of disrupting antibody-conjugated supported lipid bilayer (SLB) assemblies for the release of intact and viable CTCs from chips [40], the removal of captured cells from PLGA surfaces in intact form has yet to be exploited. Our chip system, consisting of a PLGA nanofiber arrays-coated glass cover slip and a PMMA top microfluidic plate, allowed the on-demand capture of cancer cells from whole blood samples (Fig. 6a, b). Because CTCs are rare, possibly only 1–1000 cells out of billions of blood cells from cancer patients, we spiked them at various cell densities in the whole blood of healthy individuals to explore the cell capture performance in the optimal device under the optimal operating conditions. As the number of spiked cells increased, the number of captured HCT116 cells increased linearly, with a slope and value of *R*^2^ of 0.80 and 0.9927, respectively (Fig. 6b). According to the shorter median progression-free survival for cancer patients in a training set, it would be reasonable to predict a level of CTCs equal to or higher than five per 7.5 mL of whole blood. Therefore, the high cell-capture performance of our PLGA chip system suggests its potential use for CTC isolation, similar to that of CellSearch, the only currently FDA-approved system. In addition, when integrated with the air foam technique using a 250-μL bubble solution, our PLGA-based nanofiber microfluidic system provides a recovery rate compatible with that of the SLB system when testing 200 spiked cells in 2 mL of blood from healthy individuals (Fig. 6c, d). When the medium bubble was driven at a flow rate of 5 mL h^−1^ for 10 min, Fig. 6d reveals a recovery rate of 48.5% for aligned PLGA nanofibers; the recovery rate was 72.5% on the random PLGA nanofiber arrays—the same performance as that of the SLB system. Although we do not fully understand the mechanism behind using air bubbles to release cells bound to EpCAM-conjugated PLGA nanofibers, it appears that our air foam technology might be a universal method compatible with the PLGA-based NanoVelcro platform for use in future cell-recovery applications (Fig. 6d).

To further examine the differences in the cell-capture and -release performances when using our random and aligned PLGA nanofiber arrays, we employed tapping-mode AFM to identify their individual topographical characteristics (Additional file 1: Figure S2). The root-mean-square roughness (*R*_rms_) of the random PLGA nanofibers (ca. 1.01 μm) was greater than that of the aligned PLGA nanofibers (ca. 0.47 μm). It is believed that the random PLGA nanofibers may have more cell attachments than aligned because of their more realistic cell-fiber interactions, thereby allowing cancer cells firmly trapped into the networks of nanofibers. Additionally, the differences in the capture yields and recovery rates of the CTCs when using our microfluidic system can be ascribed to the surface roughness of the PLGA nanofiber arrays in the microfluidic channels, with greater roughness promoting the contact frequency at the cell–substrate interface and the contact interaction at the air foam–substrate interface. We attribute the high cell capture and release efficiencies of the CTCs on the random nanofibers to the injected liquid biopsy tending to result in a more turbulent flow when passing through a rougher surface, as well as the creation of a higher shear stress for the nondestructive removal of more captured cells from the PLGA surfaces.

**Fig. 7:**
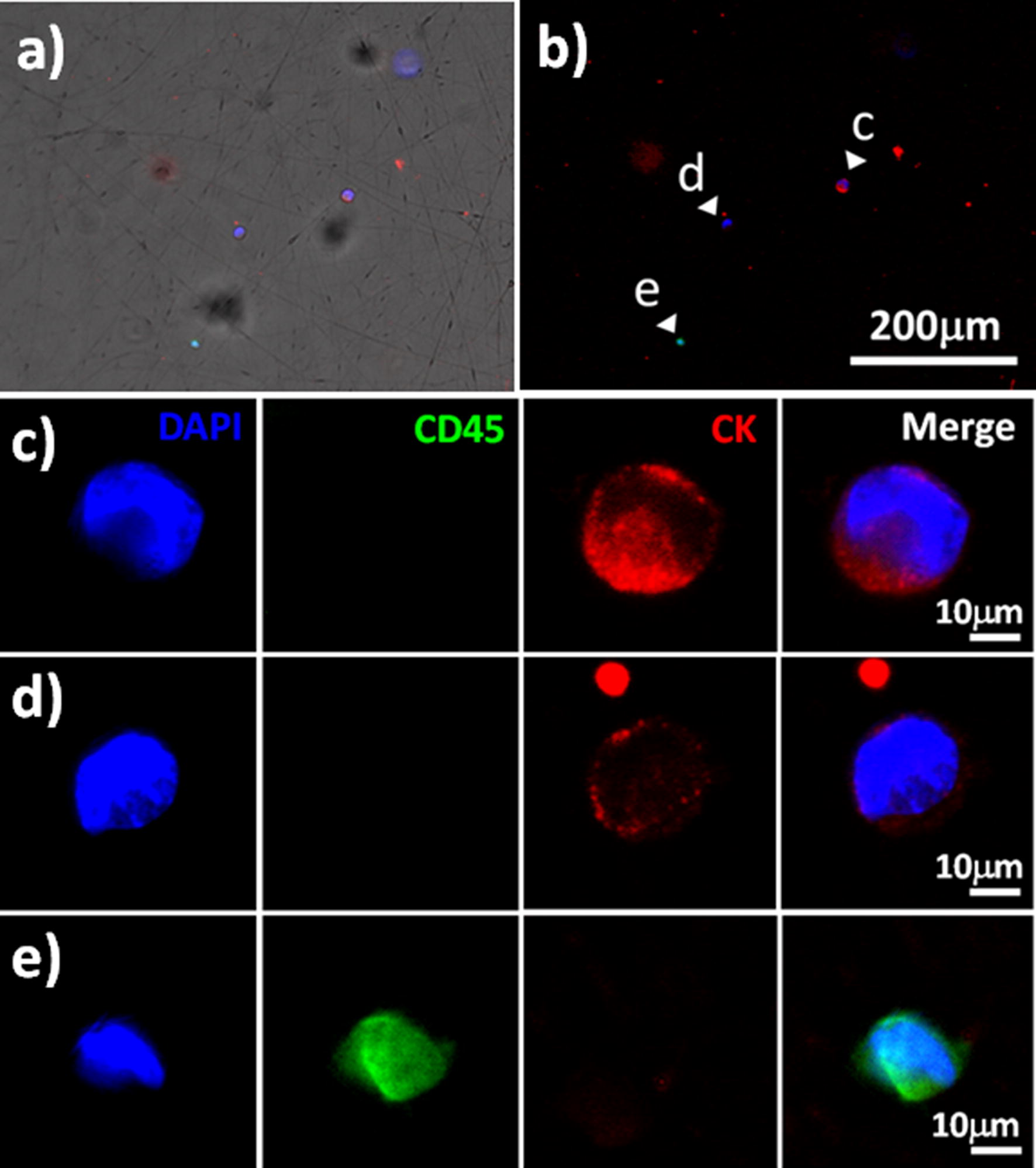
Immuno-fluorescent images of spiked HCT116 cells captured on the PLGA nanofiber arrays chips from a whole blood sample; DAPI staining for nuclei; CK staining for epithelial cancer cells; CD45 staining for white blood cells. **a** Merged fluorescence image. **b** Fluorescence image, **c**, **d** HCT116 cells. **e** White blood cells from healthy individuals

Fluorescence information is critical when differentiating cell types or cell viabilities after the cell-capture process. Notably, the optical transparency of our PLGA nanofiber-based chip system is sufficiently high for the direct bioimaging of captured cells through an inverted microscope. After passing artificial blood biopsies through our optimized device system, the captured cells specific for the anti-EpCAM antibodies were washed with PBS to eliminate any unbound cells from the device; the captured cells were further confirmed by staining with DAPI to observe the nuclei, and with CD45 and cytokeratin 20 (CK20) for the specific immunofluorescence of WBCs and HCT116, respectively. Figure 7a, b present spatial images of the fluorescent-stained cells distributed on the PLGA nanofiber surfaces. In Fig. 7c–e, the cells were stained with DAPI (blue) to identify the captured cells in terms of the presence of their nuclei; Fig. 7e reveals the cells stained with CD45 antigens, with a fluorescent green color specific for WBCs; Fig. 7c, d display the cells that had been stained red with CK20, specific for their epithelial origin that is a molecular characteristic for CTCs. Furthermore, when comparing the cell viability with the control of tissue culture polystyrene (TCPS), Additional file 1: Figure S3 reveals that our PLGA nanofiber arrays not only provided a viability for released cells (off-chip on TCPS dishes) as high as approximately 95% but also preserved the viability of the expanding cultures (on-chip captured after 24 h of incubation).

## Conclusions

Random and aligned PLGA nanofibrous arrays are simple to fabricate on large-scales through electrospinning, and have high capabilities for dynamic isolation of CTCs when surface-modified with anti-cancer antibodies and integrated with PMMA microfluidic top plates. Our PLGA nanofiber-based microfluidic systems were capable not only of isolating spiked cancer cells from whole blood samples but also releasing the captured cells when using an air foam technology, with the released cells retaining high viability. From the viewpoint of surface modification, the EDC/NHS-mediated linking of biotin–(PEG)_7_–NH_2_ to the carboxylic acid groups on the surface of the PLGA nanofibrous arrays was confirmed through characterization and imaging using ToF–SIMS. We efficiently modified the surfaces of the PLGA nanofibers through a sequence involving successive coatings with biotin–(PEG)_7_–NH_2_, SA, and biotin-anti-EpCAM. This approach provided a synergetic effect for capturing EpCAM-positive cells while minimizing nonspecific WBC binding. For effective CTC isolation, the optimal conditions for microfluidic device preparation and operation provided almost 100% capture yield when using a high concentration of biotin–(PEG)_7_–amine (500 µg mL^−1^), a sample flow rate of 1.5 mL h^−1^, and a fiber diameter of 500 nm on the random nanofiber device, in a study of 200 EpCAM-positive HCT116 spiked cells in 2 mL of cell culture medium. In whole blood sample tests (blood from healthy individuals without pre-dilution, red blood cell lysis treatment, or any other processing), the high roughness and dense distribution of the anti-EpCAM antibodies on the random nanofiber arrays resulted in the optimal cell-capture efficiency of the HCT116 cells reaching a capture yield of 80.5% that was higher than that of the aligned-nanofiber device (65.5% capture yield). When integrated with the air foam technology, the proposed device systems provided recovery rates of 48.5% with the aligned fibers and 72.5% with the random PLGA fibers—in close concordance with the high surface roughness enhancing the cell–foam interactions and, thereby, the cell releasing performance.

## Additional file

**Additional file 1.** ToF-SIMS characterization of PEGylated biotin-conjugated PLGA nanofibers. **Figure S1.** Static cell-capture efficiencies of HCT116, MCF7, PC9, HepG2, Huh7, HeLa, and THP1 cell lines on random PLGA nanofiber arrays. HCT116, MCF7: EpCAM-positive cancer cell lines; HeLa, THP1: EpCAM-negative cancer cell lines. **Figure S2.** Three-dimensional representations of AFM topographic images and root mean square average roughnesses (R_rms_) of **a** random and **b** aligned PLGA nanofiber arrays. **Figure S3.** **a** Cell viability tests for MCF7 cells captured on the control (tissue culture polystyrene, TCPS) and through on-chip (random PLGA nanofiber arrays) and off-chip cell collection (MCF7 cells released from random PLGA nanofiber arrays using the air foam technology). Released cells were washed twice with PBS and incubated for 24 h. Viability was assayed through the fluorescence live/dead staining result, which showed calcein AM (green) for live cells and Eth-1 (red) for dead cells (*N* = 3). **b** Live/dead staining image of off-chip cell collection (incubated on TCPS for 3 h).

## References

[1] Massague J (2016). Metastatic colonization by circulating tumour cells. Nature.

[2] Mohme M (2016). Circulating and disseminated tumour cells: mechanisms of immune surveillance and escape. Nat Rev Clin Oncol..

[3] Wit SD (2015). The detection of EpCAM+ and EpCAM− circulating tumor cells. Sci Rep..

[4] Lu Y (2016). Bioresponsive materials. Nat Rev Mater..

[5] Pilipchuk SP (2015). Tissue engineering for bone regeneration and osseointegration in the oral cavity. Dent Mater.

[6] Cui W (2010). Electrospun nanofibrous materials for tissue engineering and drug delivery. Sci Technol Adv Mater..

[7] Ru C (2015). Suspended, shrinkage-free, electrospun PLGA nanofibrous scaffold for skin tissue engineering. ACS Appl Mater Interfaces..

[8] Zhang E (2016). Electrospun PDLLA/PLGA composite membranes for potential application in guided tissue regeneration. Mater Sci Eng C Mater Biol Appl..

[9] Feng C (2010). Recent progress in the preparation, characterization, and applications of nanofibers and nanofiber membranes via electrospinning/interfacial polymerization. J Appl Polym Sci.

[10] Lu Y (2014). Characterization and cytotoxicity study of nanofibrous mats incorporating rectorite and carbon. RSC Adv..

[11] Zhan Y (2015). Construction of lysozyme exfoliated rectorite-based electrospun nanofibrous membranes for bacterial inhibition. J Appl Polym Sci.

[12] Lamboni L (2015). Silk sericin: a versatile material for tissue engineering and drug delivery. Biotechnol Adv.

[13] Xin S (2017). Recyclable *Saccharomyces cerevisiae* loaded nanofibrous mats with sandwich structure constructing. J Hazard Mater.

[14] Cheng G (2018). Advanced silk fibroin biomaterials for cartilage regeneration. ACS Biomater Sci Eng..

[15] Chen J (2018). Chitosan/silk fibroin modified nanofibrous patches with mesenchymal stem cells prevent heart. Acta Biomater.

[16] Mehrasa M (2015). Electrospun aligned PLGA and PLGA/gelatin nanofibers embedded with silica nanoparticles for tissue engineering. Int J Biol Macromol.

[17] Mo Y (2015). Preparation and properties of PLGA nanofiber membranes reinforced with cellulose nanocrystals. Colloid Surf B Biointerfaces.

[18] Ranjbar-Mohammadi M (2016). Electrospinning of PLGA/gum tragacanth nanofibers containing tetracycline hydrochloride for periodontal regeneration. Mater Sci Eng C Mater Biol Appl..

[19] Khan M (2015). Evaluation of changes in morphology and function of human induced pluripotent stem cell derived cardiomyocytes (HiPSC-CMs) cultured on an aligned-nanofiber cardiac patch. PLoS ONE.

[20] Andree KC (2016). Capture of tumor cells on anti-EpCAM-functionalized poly(acrylic acid)-coated surfaces. ACS Appl Mater Interfaces.

[21] Kim YJ (2016). Poly(ethylene glycol)-modified tapered-slit membrane filter for efficient release of captured viable circulating tumor cells. Anal Chem.

[22] Meunier A (2016). Combination of mechanical and molecular filtration for enhanced enrichment of circulating tumor cells. Anal Chem.

[23] Ranjbar-Mohammadi M (2016). Electrospun curcumin loaded poly(ε-caprolactone)/gum tragacanth nanofibers for biomedical application. Int J Biol Macromol.

[24] Ebrahimi-Barough S (2015). Evaluation of motor neuron-like cell differentiation of hEnSCs on biodegradable PLGA nanofiber scaffolds. Mol Neurobiol.

[25] Vashisth P (2015). Antiproliferative activity of ferulic acid-encapsulated electrospun PLGA/PEO nanofibers against MCF-7 human breast carcinoma cells. 3 Biotech..

[26] Ma L (2015). Trap effect of three-dimensional fibers network for high efficient cancer-cell capture. Adv Healthc Mater.

[27] Massumi M (2011). The effect of topography on differentiation fates of matrigel-coated mouse embryonic stem cells cultured on PLGA nanofibrous scaffolds. Tissue Eng Part A.

[28] Zhao L (2013). High-purity prostate circulating tumor cell isolation by a polymer nanofiber-embedded microchip for whole exome sequencing. Adv Mater.

[29] Zhao Y (2015). Capturing hepatocellular carcinoma cells using lactobionic acid-functionalized electrospun polyvinyl alcohol/polyethyleneimine nanofibers. RSC Adv..

[30] Hou S (2013). Polymer nanofiber-embedded microchips for detection, isolation, and molecular analysis of single circulating melanoma cells. Angew Chem Int Ed.

[31] Sfakis L (2016). Core/shell nanofiber characterization by Raman scanning microscopy. Biomed Opt Express..

[32] Sreerekha PR (2013). Fabrication of electrospun poly(lactide-*co*-glycolide)-fibrin multiscale scaffold for myocardial regeneration in vitro. Tissue Eng Part A.

[33] Hall Barrientos IJ (2016). Fabrication and characterisation of drug-loaded electrospun polymeric nanofibers for controlled release in hernia repair. Int J Pharm.

[34] Chen JY (2016). Sensitive and specific biomimetic lipid coated microfluidics to isolate viable circulating tumor cells and microemboli for cancer detection. PLoS ONE.

[35] Gabriel MT (2016). Circulating tumor cells: a review of non-EpCAM-based approaches for cell enrichment and isolation. Clin Chem.

[36] Fischer MJ, Mol NJ, Fischer MJ (2010). Amine coupling through EDC/NHS: a practical approach. Surface plasmon resonance.

[37] Liu EY (2016). Improved protein conjugation with uniform, macroporous poly(acrylamide-*co*-acrylic acid) hydrogel microspheres via EDC/NHS chemistry. Langmuir.

[38] Jeon S (2014). High-purity isolation and recovery of circulating tumor cells using conducting polymer-deposited microfluidic device. Theranostics..

[39] Brinkmann F (2015). A versatile microarray platform for capturing rare cells. Sci Rep.

[40] Lai JM (2014). Efficient elusion of viable adhesive cells from a microfluidic system by air foam. Biomicrofluidics..

[41] Zhang N (2012). Electrospun TiO_2_ nanofiber-based cell capture assay for detecting circulating tumor cells from colorectal and gastric cancer patients. Adv Mater.

[42] Hsiao YS (2014). 3D bioelectronic interface: capturing circulating tumor cells onto conducting polymer-based micro/nanorod arrays with chemical and topographical control. Small.

[43] Hsiao YS (2015). Integrated 3D conducting polymer-based bioelectronics for capture and release of circulating tumor cells. J Mater Chem B.

[44] Chen PJ (2017). Self-assembled coronene nanofiber arrays: toward integrated organic bioelectronics for efficient isolation, detection, and recovery of cancer cells. RSC Adv..

[45] Yu CC (2017). Poly(3,4-ethylenedioxythiophene)-based nanofiber mats as an organic bioelectronic platform for programming multiple capture/release cycles of circulating tumor cells. ACS Appl Mater Interfaces.

[46] Lee AW (2018). Binary-blend fibber-based capture assay of circulating tumor cells for clinical diagnosis of colorectal cancer. J Nanobiotechnol.

[47] Yu L (2017). High throughput preparation of aligned nanofibers using an improved bubble-electrospinning. Polymers..

[48] Armbrecht L (2017). Recent advances in the analysis of single cells. Anal Chem.

[49] Jan YJ (2018). NanoVelcro rare-cell assays for detection and characterization of circulating tumor cells. Adv Drug Deliv Rev.

[50] Shen MY (2018). Glycan stimulation enables purification of prostate cancer circulating tumor cells on PEDOT NanoVelcro chips for RNA biomarker detection. Adv Healthc Mater.

[51] Hou S (2017). Imprinted nanovelcro microchips for isolation and characterization of circulating fetal trophoblasts: toward noninvasive prenatal diagnostics. ACS Nano.

[52] Li Y (2016). Epithelial cell adhesion molecule in human hepatocellular carcinoma cell lines: a target of chemoresistence. BMC Cancer..

