# Supplementary material for: Random and aligned electrospun PLGA nanofibers embedded in microfluidic chips for cancer cell isolation and integration with air foam technology for cell release
Source: J Nanobiotechnology. 2019 Feb 19;17:31. doi: 10.1186/s12951-019-0466-2 (PMC6379968; doi:10.1186/s12951-019-0466-2)
Supplement: Supplementary file 1 — Additional file 1. ToF-SIMS characterization of PEGylated biotin-conjugated PLGA nanofibers. Figure S1. Static cell-capture efficiencies of HCT116, MCF7, PC9, HepG2, Huh7, HeLa, and THP1 cell lines on random PLGA nanofiber arrays. HCT116, MCF7: EpCAM-positive cancer cell lines; HeLa, THP1: EpCAM-negative cancer cell lines. Figure S2. Three-dimensional representations of AFM topographic images and root mean square average roughnesses (Rrms) of a random and b aligned PLGA nanofiber arrays. Figure S3. a Cell viability tests for MCF7 cells captured on the control (tissue culture polystyrene, TCPS) and through on-chip (random PLGA nanofiber arrays) and off-chip cell collection (MCF7 cells released from random PLGA nanofiber arrays using the air foam technology). Released cells were washed twice with PBS and incubated for 24 h. Viability was assayed through the fluorescence live/dead staining result, which showed calcein AM (green) for live cells and Eth-1 (red) for dead cells (N = 3). b Live/dead staining image of off-chip cell collection (incubated on TCPS for 3 h). [file 12951_2019_466_MOESM1_ESM.docx]

[Additional Information]

**Random and Aligned Electrospun PLGA Nanofibers Embedded in Microfluidic Chips for Cancer Cell Isolation and Integration with Air Foam Technology for Cell Release**

Chia-Cheng Yu^1,2,^**^+^**, Yi-Wen Chen^2,^**^+^**, Po-Ying Yeh^3,^**^+^**, Yu-Sheng Hsiao^1,*^, Wei-Ting Lin^1^, Chiung-Wen Kuo^2^, Di-Yen Chueh^2^, Yun-Wen You^2^, Jing-Jong Shyue^2^, Ying-Chih Chang^3,*^ and Peilin Chen^2,*^

^1^ Department of Materials Engineering, Ming Chi University of Technology, Taishan, New Taipei City 24301, Taiwan.

E-mail: [yshsiao@mail.mcut.edu.tw](mailto:yshsiao@mail.mcut.edu.tw); Fax: +886-2-2908-4091

^2^ Research Center for Applied Sciences, Academia Sinica, Taipei 11529, Taiwan.

E-mail: [peilin@gate.sinica.edu.tw](mailto:peilin@gate.sinica.edu.tw); Fax: +886-2-2782-6680

^3^ Genomics Research Center, Academia Sinica, ,Taipei 11529, Taiwan.

E-mail: [yingchih@gate.sinica.edu.tw](mailto:peilin@gate.sinica.edu.tw); Fax: +886-2-2789-9931

^+^These authors contributed equally to this work.

**ToF-SIMS characterization**

The primary ion source was operated at 30 kV (direct current: 1 nA), with a 50 or 100 μm raster size at an incident angle of 40°. Bi_3_^+^ primary ions (27% of total ions) were selected using a double-pulsing system; the pulse width was 9.6 ns. The pulse rate was 7850 Hz, and the acquisition dosage was within the static limit, between 1.5 × 10^12^ and 9.2 × 10^12^ ions cm^–2^. When acquiring positively charged secondary ions, a pulsed 10-V flooding electron and Ar^+^ beam was applied between each Bi_3_^+^ pulse for charge compensation. When acquiring negatively charged secondary ions, only a pulsed 10-V flooding electron beam was used for charge compensation. The energy pass band was set at 240 eV.

**
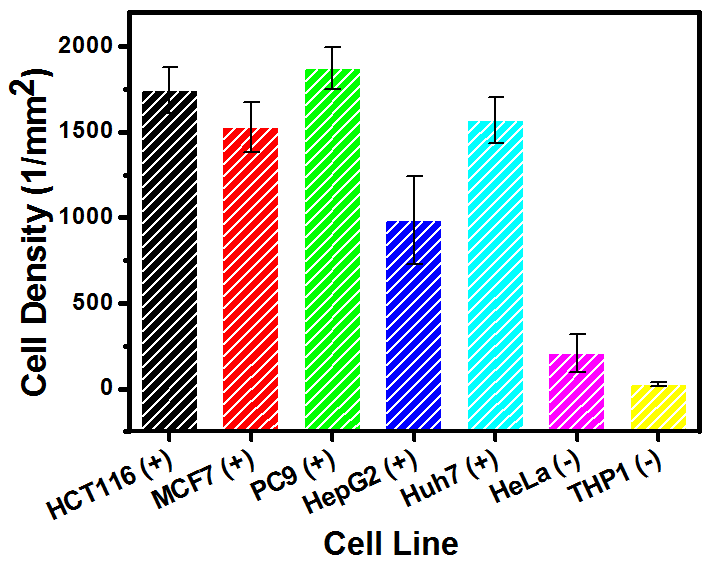
**

**Figure S1.** Static cell-capture efficiencies of HCT116, MCF7, PC9, HepG2, Huh7, HeLa, and THP1 cell lines on random PLGA nanofiber arrays. HCT116, MCF7: EpCAM-positive cancer cell lines; HeLa, THP1: EpCAM-negative cancer cell lines.


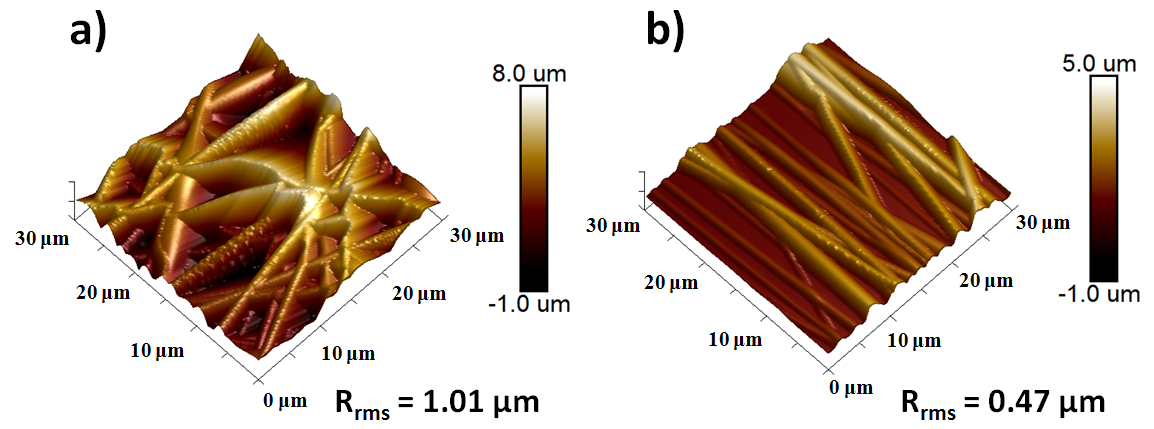


**Figure S2.** Three-dimensional representations of AFM topographic images and root mean square average roughnesses (*R*_rms_) of (a) random and (b) aligned PLGA nanofiber arrays.


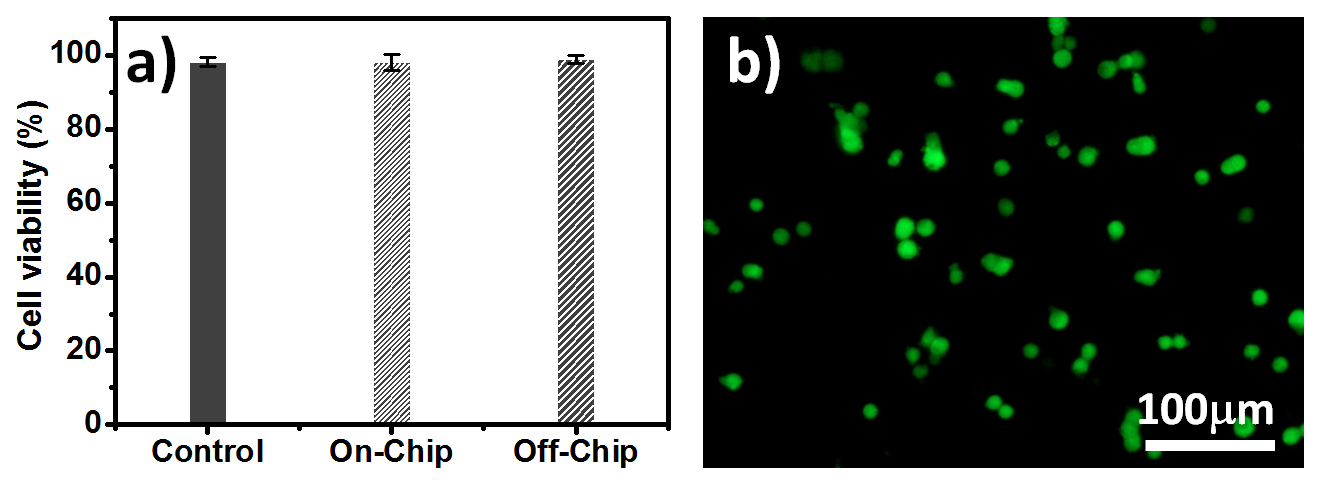


**Figure S3.** (a) Cell viability tests for MCF7 cells captured on the control (tissue culture polystyrene, TCPS) and through on-chip (random PLGA nanofiber arrays) and off-chip cell collection (MCF7 cells released from random PLGA nanofiber arrays using the air foam technology). Released cells were washed twice with PBS and incubated for 24 h. Viability was assayed through the fluorescence live/dead staining result, which showed calcein AM (green) for live cells and Eth-1 (red) for dead cells. (*N* = 3). (b) Live/dead staining image of off-chip cell collection (incubated on TCPS for 3 h).
